# *N*-(*n*-Dec­yl)-4-nitro­aniline

**DOI:** 10.1107/S1600536808003310

**Published:** 2008-02-06

**Authors:** Matthew M. Yonkey, Christopher P. Walczak, Philip J. Squattrito, Dillip K. Mohanty, Kristin Kirschbaum

**Affiliations:** a Department of Chemistry, Central Michigan University, Mount Pleasant, Michigan 48859, USA; b Department of Chemistry, University of Toledo, Toledo, Ohio 43606-3390, USA

## Abstract

*N*-(*n*-Dec­yl)-4-nitro­aniline, C_16_H_26_N_2_O_2_, crystallizes with two essentially planar mol­ecules in the asymmetric unit. The decyl chains are fully extended in an anti conformation. The mol­ecules pack in planar layers, within which mol­ecules are linked into chains by two approximately linear N—H⋯O hydrogen bonds between the amine N atom and one O atom of the nitro group of an adjacent mol­ecule. These mol­ecular chains propagate *via* inter­leaving of the decyl chains to form the two dimensional sheets. The sheets are associated exclusively *via* non-bonded contacts. The structure has features in common with those of other *N*-alkyl-4-nitro­anilines, but also subtle differences in packing.

## Related literature

For the structures of other *N*-alkyl-4-nitro­anilines, see: Panunto *et al.* (1987 ▶); Gangopadhyay *et al.* (1999 ▶); Teng *et al.* (2006 ▶).
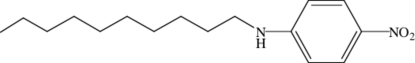


## Experimental

### 

#### Crystal data

C_16_H_26_N_2_O_2_
*M* *_r_* = 278.39
Monoclinic, 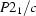

*a* = 13.291 (6) Å
*b* = 29.117 (12) Å
*c* = 8.279 (4) Å
β = 91.457 (7)°
*V* = 3203 (2) Å^3^
*Z* = 8
Mo *K*α radiation
μ = 0.08 mm^−1^
*T* = 140 (2) K
0.40 × 0.35 × 0.28 mm

#### Data collection

Bruker SMART 6000 CCD diffractometer
Absorption correction: multi-scan (*SADABS*; Sheldrick, 1996 ▶; Blessing, 1995 ▶) *T*_min_ = 0.94, *T*_max_ = 0.98
28685 measured reflections
6310 independent reflections
4359 reflections with *I* > 2σ(*I*)
*R*_int_ = 0.041

#### Refinement

*R*[*F* ^2^ > 2σ(*F* ^2^)] = 0.053
*wR*(*F* ^2^) = 0.140
*S* = 1.03
6310 reflections
569 parameters
All H-atom parameters refined
Δρ_max_ = 0.19 e Å^−3^
Δρ_min_ = −0.26 e Å^−3^

### 

Data collection: *SMART* (Bruker, 2001 ▶); cell refinement: *SAINT* (Bruker, 2003 ▶); data reduction: *SAINT*; program(s) used to solve structure: *SHELXTL* (Sheldrick, 2008 ▶); program(s) used to refine structure: *SHELXTL*; molecular graphics: *DIAMOND* (Brandenberg & Berndt, 2006 ▶) and *Crystal Maker* (Crystal Maker, 2006 ▶); software used to prepare material for publication: *SHELTXL* and local programs.

## Supplementary Material

Crystal structure: contains datablocks I, global. DOI: 10.1107/S1600536808003310/lh2595sup1.cif

Structure factors: contains datablocks I. DOI: 10.1107/S1600536808003310/lh2595Isup2.hkl

Additional supplementary materials:  crystallographic information; 3D view; checkCIF report

## Figures and Tables

**Table 1:** Hydrogen-bond geometry (Å, °)

*D*—H⋯*A*	*D*—H	H⋯*A*	*D*⋯*A*	*D*—H⋯*A*
N2—H2*N*⋯O3^i^	0.873 (18)	2.234 (19)	3.101 (2)	171.7 (16)
N4—H4*N*⋯O1^ii^	0.869 (19)	2.265 (19)	3.121 (2)	168.3 (17)

Symmetry codes: (i) 
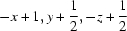
; (ii) 
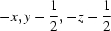
.

**Fig. 1.**
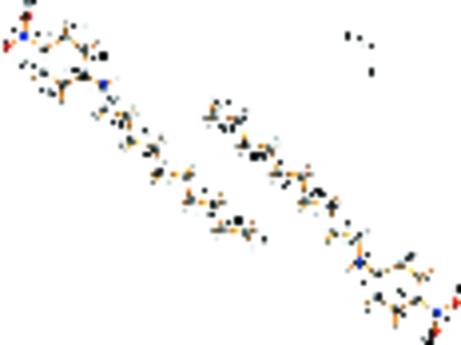
The asymmetric unit of (I) showing two independent molecules with atom labels and 50% probability displacement ellipsoids for non-hydrogen atoms.

**Fig. 2.**
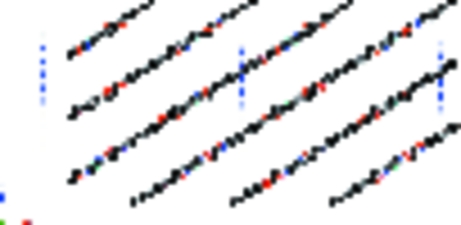
Packing diagram of (I) as viewed along the b axis. Note the planar layers parallel to (202).

**Fig. 3.**
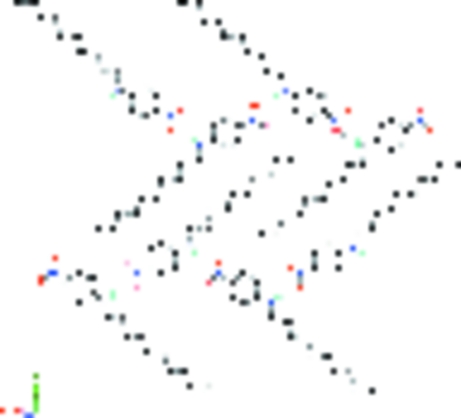
View onto a single layer of molecules with hydrogen bonds shown as dashed lines.

## supplementary crystallographic information

### Comment

Recently we have reported the preparation and characteristics of highly solvent-resistant polyamines containing two aromatic nitro groups in the polymer repeat units (Teng *et al.*, 2006). These polymers were prepared by the nucleophilic displacement of fluorine atoms from 1,5-difluoro-2,4-dinitrobenzene, using aliphatic diamines. The aforementioned solvent resistance of these polymers is a direct consequence of inter- and intrachain hydrogen bonding interactions between the secondary amine and nitro groups in the polymer repeat units. In order to further understand the importance of these interactions, we have undertaken the preparation of polyamines containing one aromatic nitro group in contrast to two such groups. The syntheses and properties of these polymers using both 2,6-difluoronitrobenzene and isomeric 2,4-difluoronitrobenzene, and a series of aliphatic diamines, will be reported elsewhere. In the course of these studies, it was necessary to prepare model compounds prior to the preparation of the polymers. This was accomplished by the reactions of either mono- or difluoro-substituted nitrobenzene with a homologous series of n-alkyl amines. The X-ray crystal structures of *N*-methyl-4-nitroaniline (Panunto *et al.*, 1987) and *N*-alkyl-4-nitroanilines with alkyl groups ranging from n-propyl to n-pentyl (Gangopadhyay *et al.*, 1999) have been reported. The *N*-decyl-4-nitroaniline (I) reported herein represents the longest chain *N*-alkyl-4-nitroaniline derivative thus far characterized by single-crystal X-ray methods.

The asymmetric unit of the title compound (I) contains two independent molecules which do not differ significantly in conformation (Figure 1). Both molecules are essentially planar and have the decyl tails in the characteristic zigzag anti conformations. The molecules are packed into layers that are parallel to and at the same intervals as the (202) lattice planes (Figure 2). Within each of these layers, the molecules are linked by two N—H···O hydrogen bonds between the amine N atom of one molecule and one of the nitro O atoms of another (Figure 3). As a consequence of these interactions, the N—O bond that participates in the hydrogen bond is about 0.01 Å longer than the other N—O bond on each nitro group. Also, the relative shortness of the H···O interactions and the linearity of the N—H···O bonds (Table 1) clearly demonstrate that these are classic single-acceptor hydrogen bonds, unlike the three-center interactions found in many nitroaniline derivatives including *N*-methyl-4-nitroaniline (Panunto *et al.*, 1987). The hydrogen bonding links the molecules into chains that run along the 101 direction with the decyl chains on adjacent molecules oriented up and down, with an angle between the chains of about 77°. To complete the two-dimensional layers, these molecular chains then stack along the *b* axis with the decyl tails interleaved in parallel fashion so as to maximize favorable non-bonded contacts.

The series of *N*-alkyl-4-nitroanilines where alkyl = propyl-octyl has been examined for potential optical second harmonic generation (SHG) behavior (Gangopadhyay *et al.*, 1999). SHG effects require the absence of a center of inversion, although this condition alone does not guarantee activity. Physical measurements of this series showed that only the butyl compound is active, and single-crystal structure analyses of the propyl, butyl and pentyl derivatives confirmed that only the butyl crystallizes in an noncentrosymmetric space group. The authors report that poor crystal quality prevented structure determinations of the longer chain derivatives, however, our successful crystallization of the decyl compound demonstrates that at least some of these may be characterized. The N—H···O hydrogen bonding in (I) is very similar to that found in the shorter chain analogs in terms of involving only one of the nitro O atoms. The packing in (I) is subtly different. In the propyl and pentyl compounds, the molecules stack in one direction in identical orientation so that both the rings and chains are parallel and in close contact. By contrast, in (I), the layers are staggered so that rings in adjacent layers are not directly over one another but rather have an alkyl chain in between. This indicates that the fully interleaved packing of the decyl chains within the layer, which is unique to (I), is more important than the π-π interactions between the rings in determining the overall packing.

### Experimental

Anhydrous potassium carbonate (2.0811 g, 0.015 mol) and a solution of 4-nitrofluorobenzene (0.9993 g, 0.007 mol) in 8 ml of dimethyacetamide (DMAC) were combined in a three-necked 100 ml round-bottomed flask, fitted with a nitrogen inlet, a thermometer, a magnetic stirring bar, and a Dean-Stark trap fitted with a condenser. To the clear yellow solution, n-decylamine (1.1767 g, 0.0075 mol) dissolved in DMAC (5 ml) was added with stirring. Additional DMAC (8 ml) was used to wash the transfer container and this was added to the reaction mixture, followed by the addition of 20 ml of toluene. The temperature of the reaction mixture was raised to 403 K, and the reaction was allowed to continue at this temperature for one hour. Water, the by-product of the reaction, was removed *via* azeotropic distillation with toluene. After the removal of water, toluene was removed *via* the Dean-Stark trap, and the temperature of the reaction mixture was allowed to rise to 433 K. The reaction was allowed to continue at this temperature for three hours, after which it was allowed to cool to room temperature and then diluted with 20 ml of dichloromethane. The resulting heterogeneous mixture was then filtered through celite at reduced pressure, and the solvents from the filtrate were removed under high vacuum to yield a bright orange liquid residue. This crude product was dissolved in trichloromethane (15 ml), transferred to a separatory funnel, and washed repeatedly with deionized water. The organic layer was collected, dried over anhydrous magnesium sulfate, filtered, and the filtrate was evaporated using a rotary evaporator to yield a bright yellow solid. Crystals suitable for X-ray diffraction were obtained by recrystallization from hexane. Yield 56%, m.p. 332–333 K. IR (KBr, ν > 1400 cm^-1^): 3353, 3064, 2952, 2925, 2850, 1602, 1541, 1475, 1466. ^1^H NMR [400 MHz, δ p.p.m., CDCl_3_], 8.12 (m, 2H), 6.53 (m, 2H), 4.44 (s, 1H), 1.65 (m, 2H), 1.30 (m, 14H), 0.92 (t, 3H). ^13^C NMR [δ, CDCl_3_], 153.63, 137.74, 129.69.111.11, 43.66, 32.09, 29.75, 29.54, 29.51, 29.36, 27.21, 22.89, 14.33. MS (*M*/*Z*) (% base peak), 278 (11.6), 151 (100), 105 (19.7).

### Refinement

Upon evaluation of systematic absences and weaknesses, the space group was determined to be *P*2_1_/*c* with an additional pseudo-a glide perpendicular to the *b* axis. A partial structure solution was obtained by direct methods and revealed two crystallographically independent molecules in the asymmetric unit. The remaining non-hydrogen atoms were located with difference Fourier techniques and refined with anisotropic atomic displacement parameters. All hydrogen atoms could be located in the difference Fourier maps and refined isotropically.

### Crystal data

**Table:**

C_16_H_26_N_2_O_2_	*F*_000_ = 1216
*M**_r_* = 278.39	*D*_x_ = 1.155 Mg m^−^^3^
Monoclinic, *P*2_1_/*c*	Melting point = 332–333 K
Hall symbol: -P 2ybc	Mo *K*α radiation λ = 0.71073 Å
*a* = 13.291 (6) Å	Cell parameters from 950 reflections
*b* = 29.117 (12) Å	θ = 2.6–25.8º
*c* = 8.279 (4) Å	µ = 0.08 mm^−^^1^
β = 91.457 (7)º	*T* = 140 (2) K
*V* = 3203 (2) Å^3^	Pyramidal, yellow
*Z* = 8	0.40 × 0.35 × 0.28 mm

### Data collection

**Table:**

Bruker SMART 6000 CCD diffractometer	6310 independent reflections
Radiation source: fine-focus sealed tube	4359 reflections with *I* > 2σ(*I*)
Monochromator: graphite	*R*_int_ = 0.041
*T* = 140(2) K	θ_max_ = 26.1º
ω scans	θ_min_ = 1.5º
Absorption correction: multi-scan(SADABS; Sheldrick, 1996; Blessing, 1995)	*h* = −16→16
*T*_min_ = 0.94, *T*_max_ = 0.98	*k* = −32→35
28685 measured reflections	*l* = −10→10

### Refinement

**Table:**

Refinement on *F*^2^	Secondary atom site location: difference Fourier map
Least-squares matrix: full	Hydrogen site location: difference Fourier map
*R*[*F*^2^ > 2σ(*F*^2^)] = 0.053	All H-atom parameters refined
*wR*(*F*^2^) = 0.140	*w* = 1/[σ^2^(*F*_o_^2^) + (0.0796*P*)^2^ + 0.231*P*] where *P* = (*F*_o_^2^ + 2*F*_c_^2^)/3
*S* = 1.03	(Δ/σ)_max_ = 0.001
6310 reflections	Δρ_max_ = 0.19 e Å^−^^3^
569 parameters	Δρ_min_ = −0.26 e Å^−^^3^
Primary atom site location: structure-invariant direct methods	Extinction correction: none

### Special details

**Table:**

Geometry. All e.s.d.'s (except the e.s.d. in the dihedral angle between two l.s. planes) are estimated using the full covariance matrix. The cell e.s.d.'s are taken into account individually in the estimation of e.s.d.'s in distances, angles and torsion angles; correlations between e.s.d.'s in cell parameters are only used when they are defined by crystal symmetry. An approximate (isotropic) treatment of cell e.s.d.'s is used for estimating e.s.d.'s involving l.s. planes.
Refinement. Refinement of *F*^2^ against ALL reflections. The weighted *R*-factor *wR* and goodness of fit *S* are based on *F*^2^, conventional *R*-factors *R* are based on *F*, with *F* set to zero for negative *F*^2^. The threshold expression of *F*^2^ > σ(*F*^2^) is used only for calculating *R*-factors(gt) *etc*. and is not relevant to the choice of reflections for refinement. *R*-factors based on *F*^2^ are statistically about twice as large as those based on *F*, and *R*- factors based on ALL data will be even larger.

### Fractional atomic coordinates and isotropic or equivalent isotropic displacement parameters (Å^2^)

**Table:**

	*x*	*y*	*z*	*U*_iso_*/*U*_eq_	
O1	−0.60160 (9)	0.67377 (4)	−0.86284 (15)	0.0412 (3)	
O2	−0.67258 (9)	0.60818 (4)	−0.90706 (16)	0.0453 (4)	
O3	1.10779 (9)	0.09460 (4)	0.85523 (15)	0.0406 (3)	
O4	1.17105 (10)	0.16069 (4)	0.91774 (17)	0.0484 (4)	
N1	−0.60284 (10)	0.63137 (5)	−0.85126 (16)	0.0302 (3)	
N2	−0.27827 (10)	0.54358 (5)	−0.54454 (16)	0.0295 (3)	
N3	1.10435 (10)	0.13712 (5)	0.85407 (16)	0.0320 (3)	
N4	0.77760 (11)	0.22343 (5)	0.54846 (17)	0.0312 (3)	
C1	−0.51989 (11)	0.60874 (6)	−0.77006 (18)	0.0256 (4)	
C2	−0.51950 (12)	0.56106 (6)	−0.75980 (19)	0.0276 (4)	
C3	−0.44015 (12)	0.53898 (6)	−0.68323 (19)	0.0265 (4)	
C4	−0.35872 (11)	0.56424 (5)	−0.61648 (18)	0.0246 (4)	
C5	−0.36234 (12)	0.61265 (6)	−0.62760 (19)	0.0274 (4)	
C6	−0.44100 (12)	0.63459 (6)	−0.70329 (19)	0.0273 (4)	
C7	−0.26796 (13)	0.49425 (6)	−0.5227 (2)	0.0283 (4)	
C8	−0.16565 (13)	0.48362 (6)	−0.4446 (2)	0.0300 (4)	
C9	−0.14962 (13)	0.43315 (6)	−0.4047 (2)	0.0295 (4)	
C10	−0.04873 (13)	0.42560 (6)	−0.3167 (2)	0.0317 (4)	
C11	−0.02331 (13)	0.37619 (6)	−0.2754 (2)	0.0292 (4)	
C12	0.07786 (13)	0.37202 (6)	−0.1859 (2)	0.0296 (4)	
C13	0.10972 (13)	0.32300 (6)	−0.1464 (2)	0.0290 (4)	
C14	0.20989 (13)	0.31956 (6)	−0.0544 (2)	0.0310 (4)	
C15	0.24269 (14)	0.27065 (6)	−0.0180 (2)	0.0354 (4)	
C16	0.34653 (16)	0.26753 (8)	0.0615 (3)	0.0499 (6)	
C17	1.01992 (12)	0.15945 (6)	0.77582 (18)	0.0271 (4)	
C18	1.01434 (12)	0.20703 (6)	0.77712 (19)	0.0289 (4)	
C19	0.93436 (12)	0.22876 (6)	0.70194 (19)	0.0289 (4)	
C20	0.85730 (11)	0.20307 (6)	0.62333 (18)	0.0264 (4)	
C21	0.86534 (12)	0.15453 (6)	0.62463 (19)	0.0283 (4)	
C22	0.94510 (12)	0.13317 (6)	0.69935 (19)	0.0275 (4)	
C23	0.76177 (13)	0.27270 (6)	0.5393 (2)	0.0295 (4)	
C24	0.66360 (13)	0.28244 (6)	0.4467 (2)	0.0312 (4)	
C25	0.64562 (13)	0.33306 (6)	0.4121 (2)	0.0297 (4)	
C26	0.54838 (13)	0.34037 (6)	0.3138 (2)	0.0310 (4)	
C27	0.52284 (13)	0.39032 (6)	0.2770 (2)	0.0307 (4)	
C28	0.42175 (13)	0.39529 (6)	0.1880 (2)	0.0294 (4)	
C29	0.39176 (13)	0.44438 (6)	0.1484 (2)	0.0286 (4)	
C30	0.29031 (13)	0.44809 (6)	0.0593 (2)	0.0292 (4)	
C31	0.26026 (14)	0.49683 (6)	0.0147 (2)	0.0323 (4)	
C32	0.15712 (16)	0.49976 (7)	−0.0678 (3)	0.0420 (5)	
H2N	−0.2325 (13)	0.5606 (6)	−0.496 (2)	0.032 (5)*	
H2	−0.5723 (13)	0.5437 (6)	−0.8041 (19)	0.027 (4)*	
H3	−0.4412 (13)	0.5071 (7)	−0.679 (2)	0.035 (5)*	
H4N	0.7319 (14)	0.2059 (7)	0.504 (2)	0.037 (5)*	
H5	−0.3087 (13)	0.6296 (6)	−0.5867 (19)	0.030 (5)*	
H6	−0.4425 (13)	0.6666 (7)	−0.7100 (19)	0.031 (5)*	
H7B	−0.3219 (13)	0.4826 (6)	−0.453 (2)	0.031 (4)*	
H7A	−0.2746 (12)	0.4790 (6)	−0.630 (2)	0.027 (4)*	
H8B	−0.1144 (13)	0.4933 (6)	−0.519 (2)	0.034 (5)*	
H8A	−0.1593 (13)	0.5016 (6)	−0.347 (2)	0.039 (5)*	
H9B	−0.2026 (13)	0.4221 (6)	−0.334 (2)	0.035 (5)*	
H9A	−0.1528 (12)	0.4146 (6)	−0.507 (2)	0.033 (5)*	
H10B	0.0036 (13)	0.4388 (6)	−0.385 (2)	0.034 (5)*	
H10A	−0.0496 (13)	0.4451 (6)	−0.220 (2)	0.038 (5)*	
H11B	−0.0757 (13)	0.3637 (6)	−0.205 (2)	0.030 (4)*	
H11A	−0.0216 (12)	0.3566 (6)	−0.376 (2)	0.033 (5)*	
H12B	0.1303 (13)	0.3863 (6)	−0.251 (2)	0.035 (5)*	
H12A	0.0726 (12)	0.3896 (6)	−0.083 (2)	0.031 (4)*	
H13B	0.0577 (13)	0.3094 (6)	−0.083 (2)	0.033 (5)*	
H13A	0.1142 (12)	0.3066 (6)	−0.246 (2)	0.036 (5)*	
H14B	0.2036 (12)	0.3372 (6)	0.047 (2)	0.031 (4)*	
H14A	0.2602 (14)	0.3339 (6)	−0.121 (2)	0.044 (5)*	
H15B	0.2401 (15)	0.2530 (7)	−0.116 (3)	0.054 (6)*	
H15A	0.1955 (14)	0.2567 (6)	0.052 (2)	0.039 (5)*	
H16C	0.3993 (16)	0.2807 (8)	−0.007 (3)	0.064 (7)*	
H16B	0.3464 (18)	0.2856 (9)	0.166 (3)	0.079 (8)*	
H16A	0.3665 (16)	0.2361 (9)	0.085 (3)	0.063 (7)*	
H18	1.0663 (14)	0.2236 (6)	0.829 (2)	0.037 (5)*	
H19	0.9313 (13)	0.2604 (7)	0.705 (2)	0.038 (5)*	
H21	0.8145 (13)	0.1366 (6)	0.578 (2)	0.036 (5)*	
H22	0.9501 (12)	0.1018 (6)	0.6989 (18)	0.021 (4)*	
H23A	0.7597 (13)	0.2854 (6)	0.650 (2)	0.035 (5)*	
H23B	0.8176 (12)	0.2866 (6)	0.4814 (19)	0.026 (4)*	
H24A	0.6678 (13)	0.2668 (6)	0.346 (2)	0.035 (5)*	
H24B	0.6091 (13)	0.2712 (6)	0.511 (2)	0.037 (5)*	
H25A	0.6444 (12)	0.3504 (6)	0.512 (2)	0.029 (4)*	
H25B	0.7028 (14)	0.3458 (6)	0.351 (2)	0.041 (5)*	
H26B	0.5542 (13)	0.3223 (6)	0.212 (2)	0.038 (5)*	
H26A	0.4937 (14)	0.3258 (6)	0.375 (2)	0.039 (5)*	
H27A	0.5216 (13)	0.4080 (6)	0.380 (2)	0.035 (5)*	
H27B	0.5763 (14)	0.4048 (6)	0.210 (2)	0.035 (5)*	
H28B	0.3706 (13)	0.3813 (6)	0.257 (2)	0.032 (5)*	
H28A	0.4228 (13)	0.3761 (6)	0.087 (2)	0.037 (5)*	
H29B	0.4433 (13)	0.4581 (6)	0.0850 (19)	0.034 (5)*	
H29A	0.3906 (12)	0.4617 (6)	0.252 (2)	0.030 (4)*	
H30B	0.2386 (13)	0.4343 (6)	0.131 (2)	0.033 (5)*	
H30A	0.2948 (13)	0.4291 (6)	−0.039 (2)	0.034 (5)*	
H31B	0.2590 (13)	0.5154 (6)	0.110 (2)	0.031 (4)*	
H31A	0.3098 (14)	0.5093 (7)	−0.057 (2)	0.045 (5)*	
H32C	0.1556 (15)	0.4806 (7)	−0.168 (2)	0.054 (6)*	
H32B	0.1019 (17)	0.4875 (8)	0.003 (3)	0.066 (7)*	
H32A	0.1373 (15)	0.5321 (8)	−0.101 (2)	0.057 (6)*	

### Atomic displacement parameters (Å^2^)

**Table:**

	*U*^11^	*U*^22^	*U*^33^	*U*^12^	*U*^13^	*U*^23^
O1	0.0429 (8)	0.0236 (7)	0.0564 (8)	0.0058 (5)	−0.0149 (6)	0.0077 (6)
O2	0.0334 (7)	0.0367 (8)	0.0647 (9)	−0.0033 (6)	−0.0236 (6)	0.0049 (6)
O3	0.0417 (7)	0.0266 (8)	0.0528 (8)	0.0096 (6)	−0.0097 (6)	0.0047 (6)
O4	0.0381 (8)	0.0369 (8)	0.0688 (9)	−0.0005 (6)	−0.0288 (7)	0.0033 (6)
N1	0.0289 (8)	0.0277 (9)	0.0339 (7)	0.0015 (6)	−0.0054 (6)	0.0025 (6)
N2	0.0261 (8)	0.0215 (8)	0.0402 (8)	−0.0022 (6)	−0.0109 (6)	0.0010 (6)
N3	0.0307 (8)	0.0276 (9)	0.0373 (8)	0.0045 (7)	−0.0060 (6)	0.0035 (6)
N4	0.0268 (8)	0.0247 (8)	0.0416 (8)	0.0013 (6)	−0.0113 (6)	−0.0014 (6)
C1	0.0257 (8)	0.0236 (9)	0.0273 (8)	0.0026 (7)	−0.0036 (7)	0.0023 (6)
C2	0.0243 (9)	0.0242 (10)	0.0340 (9)	−0.0036 (7)	−0.0044 (7)	−0.0016 (7)
C3	0.0274 (9)	0.0175 (9)	0.0345 (9)	0.0004 (7)	−0.0048 (7)	0.0010 (7)
C4	0.0229 (8)	0.0241 (9)	0.0265 (8)	0.0007 (7)	−0.0023 (6)	0.0011 (6)
C5	0.0258 (9)	0.0252 (10)	0.0308 (9)	−0.0049 (7)	−0.0039 (7)	−0.0005 (7)
C6	0.0318 (9)	0.0195 (9)	0.0304 (8)	−0.0006 (7)	−0.0015 (7)	0.0011 (7)
C7	0.0263 (9)	0.0216 (9)	0.0367 (9)	0.0006 (7)	−0.0049 (7)	0.0015 (7)
C8	0.0287 (9)	0.0241 (9)	0.0369 (10)	0.0006 (7)	−0.0060 (8)	0.0031 (8)
C9	0.0285 (9)	0.0231 (9)	0.0366 (10)	0.0006 (7)	−0.0039 (8)	0.0009 (7)
C10	0.0305 (9)	0.0235 (10)	0.0408 (10)	0.0002 (7)	−0.0062 (8)	0.0019 (8)
C11	0.0294 (9)	0.0237 (10)	0.0343 (9)	0.0020 (7)	−0.0033 (8)	0.0017 (7)
C12	0.0300 (9)	0.0216 (9)	0.0371 (9)	−0.0001 (7)	−0.0032 (8)	0.0031 (7)
C13	0.0293 (9)	0.0222 (9)	0.0352 (9)	0.0014 (7)	−0.0028 (7)	0.0016 (7)
C14	0.0286 (9)	0.0254 (10)	0.0388 (10)	0.0008 (7)	−0.0028 (8)	0.0050 (8)
C15	0.0352 (10)	0.0241 (10)	0.0466 (11)	0.0016 (8)	−0.0081 (9)	0.0049 (8)
C16	0.0400 (12)	0.0368 (13)	0.0719 (16)	0.0049 (10)	−0.0178 (11)	0.0130 (11)
C17	0.0253 (9)	0.0258 (10)	0.0301 (8)	0.0034 (7)	−0.0029 (7)	0.0031 (7)
C18	0.0263 (9)	0.0258 (10)	0.0343 (9)	−0.0030 (7)	−0.0076 (7)	0.0007 (7)
C19	0.0300 (9)	0.0204 (10)	0.0361 (9)	0.0009 (7)	−0.0056 (7)	0.0008 (7)
C20	0.0238 (8)	0.0276 (10)	0.0277 (8)	0.0030 (7)	−0.0014 (6)	0.0017 (6)
C21	0.0260 (9)	0.0261 (10)	0.0325 (9)	−0.0031 (7)	−0.0041 (7)	−0.0024 (7)
C22	0.0312 (9)	0.0179 (10)	0.0334 (9)	0.0018 (7)	−0.0011 (7)	−0.0002 (7)
C23	0.0292 (9)	0.0250 (10)	0.0339 (9)	0.0028 (7)	−0.0046 (7)	0.0013 (7)
C24	0.0285 (9)	0.0277 (10)	0.0372 (10)	0.0030 (7)	−0.0039 (8)	−0.0004 (8)
C25	0.0291 (9)	0.0269 (10)	0.0330 (9)	0.0042 (7)	−0.0027 (7)	−0.0012 (7)
C26	0.0330 (10)	0.0255 (10)	0.0342 (9)	0.0049 (8)	−0.0056 (8)	−0.0015 (7)
C27	0.0315 (10)	0.0262 (10)	0.0342 (9)	0.0030 (7)	−0.0042 (8)	0.0003 (7)
C28	0.0325 (9)	0.0231 (9)	0.0323 (9)	0.0025 (7)	−0.0053 (7)	−0.0004 (7)
C29	0.0299 (9)	0.0234 (10)	0.0322 (9)	0.0001 (7)	−0.0041 (7)	−0.0003 (7)
C30	0.0315 (9)	0.0212 (9)	0.0344 (9)	0.0002 (7)	−0.0056 (8)	0.0004 (7)
C31	0.0352 (10)	0.0239 (10)	0.0374 (10)	0.0016 (8)	−0.0067 (8)	−0.0018 (8)
C32	0.0398 (11)	0.0317 (12)	0.0536 (12)	0.0067 (9)	−0.0155 (9)	−0.0001 (9)

### Geometric parameters (Å, °)

**Table:**

O1—N1	1.2383 (18)	C15—C16	1.516 (3)
O2—N1	1.2275 (17)	C15—H15B	0.96 (2)
O3—N3	1.2389 (19)	C15—H15A	0.954 (19)
O4—N3	1.2293 (18)	C16—H16C	0.99 (2)
N1—C1	1.437 (2)	C16—H16B	1.01 (2)
N2—C4	1.352 (2)	C16—H16A	0.97 (2)
N2—C7	1.454 (2)	C17—C18	1.387 (2)
N2—H2N	0.873 (18)	C17—C22	1.394 (2)
N3—C17	1.437 (2)	C18—C19	1.373 (2)
N4—C20	1.351 (2)	C18—H18	0.938 (19)
N4—C23	1.452 (2)	C19—C20	1.414 (2)
N4—H4N	0.869 (19)	C19—H19	0.92 (2)
C1—C2	1.391 (2)	C20—C21	1.417 (2)
C1—C6	1.394 (2)	C21—C22	1.364 (2)
C2—C3	1.376 (2)	C21—H21	0.930 (18)
C2—H2	0.933 (17)	C22—H22	0.916 (18)
C3—C4	1.410 (2)	C23—C24	1.523 (2)
C3—H3	0.930 (19)	C23—H23A	0.987 (17)
C4—C5	1.413 (2)	C23—H23B	0.982 (17)
C5—C6	1.364 (2)	C24—C25	1.519 (2)
C5—H5	0.924 (17)	C24—H24A	0.957 (18)
C6—H6	0.935 (19)	C24—H24B	0.966 (18)
C7—C8	1.523 (2)	C25—C26	1.524 (2)
C7—H7B	0.989 (17)	C25—H25A	0.970 (17)
C7—H7A	0.997 (16)	C25—H25B	0.995 (19)
C8—C9	1.520 (2)	C26—C27	1.522 (2)
C8—H8B	0.973 (18)	C26—H26B	1.000 (18)
C8—H8A	0.965 (18)	C26—H26A	0.992 (18)
C9—C10	1.526 (2)	C27—C28	1.523 (2)
C9—H9B	0.982 (18)	C27—H27A	0.999 (18)
C9—H9A	1.002 (17)	C27—H27B	1.003 (18)
C10—C11	1.515 (2)	C28—C29	1.518 (2)
C10—H10B	0.986 (17)	C28—H28B	0.988 (17)
C10—H10A	0.984 (18)	C28—H28A	1.008 (17)
C11—C12	1.524 (2)	C29—C30	1.524 (2)
C11—H11B	0.989 (17)	C29—H29B	0.961 (18)
C11—H11A	1.012 (17)	C29—H29A	0.995 (17)
C12—C13	1.522 (2)	C30—C31	1.518 (2)
C12—H12B	0.985 (18)	C30—H30B	1.003 (17)
C12—H12A	1.000 (17)	C30—H30A	0.983 (18)
C13—C14	1.520 (2)	C31—C32	1.518 (3)
C13—H13B	0.964 (17)	C31—H31B	0.957 (17)
C13—H13A	0.959 (18)	C31—H31A	0.968 (19)
C14—C15	1.517 (3)	C32—H32C	1.00 (2)
C14—H14B	0.988 (17)	C32—H32B	1.02 (2)
C14—H14A	0.973 (19)	C32—H32A	1.01 (2)
			
O2—N1—O1	122.05 (13)	C15—C16—H16C	112.1 (12)
O2—N1—C1	119.14 (14)	C15—C16—H16B	108.6 (14)
O1—N1—C1	118.81 (13)	H16C—C16—H16B	107.8 (19)
C4—N2—C7	124.44 (14)	C15—C16—H16A	112.5 (13)
C4—N2—H2N	119.0 (12)	H16C—C16—H16A	106.6 (18)
C7—N2—H2N	116.1 (12)	H16B—C16—H16A	109.1 (19)
O4—N3—O3	121.91 (14)	C18—C17—C22	120.94 (14)
O4—N3—C17	119.14 (15)	C18—C17—N3	119.32 (14)
O3—N3—C17	118.95 (14)	C22—C17—N3	119.74 (15)
C20—N4—C23	124.66 (15)	C19—C18—C17	119.84 (15)
C20—N4—H4N	118.1 (12)	C19—C18—H18	121.6 (11)
C23—N4—H4N	117.3 (12)	C17—C18—H18	118.6 (11)
C2—C1—C6	120.88 (14)	C18—C19—C20	120.58 (16)
C2—C1—N1	119.20 (14)	C18—C19—H19	118.8 (11)
C6—C1—N1	119.91 (15)	C20—C19—H19	120.6 (11)
C3—C2—C1	119.74 (15)	N4—C20—C19	121.98 (16)
C3—C2—H2	119.2 (11)	N4—C20—C21	119.95 (15)
C1—C2—H2	121.1 (10)	C19—C20—C21	118.07 (14)
C2—C3—C4	120.57 (16)	C22—C21—C20	121.05 (15)
C2—C3—H3	118.2 (11)	C22—C21—H21	118.6 (11)
C4—C3—H3	121.2 (11)	C20—C21—H21	120.3 (11)
N2—C4—C3	122.09 (15)	C21—C22—C17	119.53 (16)
N2—C4—C5	119.85 (14)	C21—C22—H22	120.5 (10)
C3—C4—C5	118.06 (14)	C17—C22—H22	119.9 (10)
C6—C5—C4	121.42 (15)	N4—C23—C24	109.36 (14)
C6—C5—H5	119.6 (11)	N4—C23—H23A	109.2 (10)
C4—C5—H5	119.0 (11)	C24—C23—H23A	110.7 (10)
C5—C6—C1	119.31 (16)	N4—C23—H23B	108.9 (10)
C5—C6—H6	120.6 (11)	C24—C23—H23B	109.0 (9)
C1—C6—H6	120.0 (11)	H23A—C23—H23B	109.7 (14)
N2—C7—C8	109.51 (13)	C25—C24—C23	113.85 (14)
N2—C7—H7B	110.0 (10)	C25—C24—H24A	108.0 (11)
C8—C7—H7B	109.6 (10)	C23—C24—H24A	106.4 (11)
N2—C7—H7A	108.8 (9)	C25—C24—H24B	108.5 (10)
C8—C7—H7A	110.3 (9)	C23—C24—H24B	107.9 (10)
H7B—C7—H7A	108.5 (13)	H24A—C24—H24B	112.3 (15)
C9—C8—C7	114.04 (14)	C24—C25—C26	111.31 (14)
C9—C8—H8B	108.7 (10)	C24—C25—H25A	110.4 (10)
C7—C8—H8B	107.6 (10)	C26—C25—H25A	110.5 (10)
C9—C8—H8A	109.4 (11)	C24—C25—H25B	109.9 (11)
C7—C8—H8A	107.7 (11)	C26—C25—H25B	108.9 (10)
H8B—C8—H8A	109.2 (15)	H25A—C25—H25B	105.6 (14)
C8—C9—C10	111.07 (14)	C27—C26—C25	114.96 (14)
C8—C9—H9B	110.3 (10)	C27—C26—H26B	110.8 (10)
C10—C9—H9B	107.6 (10)	C25—C26—H26B	107.1 (10)
C8—C9—H9A	109.6 (10)	C27—C26—H26A	110.3 (11)
C10—C9—H9A	110.0 (10)	C25—C26—H26A	106.8 (10)
H9B—C9—H9A	108.1 (14)	H26B—C26—H26A	106.4 (14)
C11—C10—C9	115.65 (14)	C26—C27—C28	112.15 (14)
C11—C10—H10B	110.1 (10)	C26—C27—H27A	109.2 (10)
C9—C10—H10B	107.1 (10)	C28—C27—H27A	109.4 (10)
C11—C10—H10A	111.7 (11)	C26—C27—H27B	110.7 (10)
C9—C10—H10A	106.2 (10)	C28—C27—H27B	108.9 (10)
H10B—C10—H10A	105.6 (14)	H27A—C27—H27B	106.4 (14)
C10—C11—C12	111.98 (14)	C29—C28—C27	114.68 (14)
C10—C11—H11B	109.0 (10)	C29—C28—H28B	109.4 (10)
C12—C11—H11B	108.0 (9)	C27—C28—H28B	106.9 (9)
C10—C11—H11A	111.0 (10)	C29—C28—H28A	110.6 (10)
C12—C11—H11A	108.6 (9)	C27—C28—H28A	108.6 (10)
H11B—C11—H11A	108.1 (14)	H28B—C28—H28A	106.2 (14)
C13—C12—C11	114.60 (14)	C28—C29—C30	113.29 (14)
C13—C12—H12B	108.4 (10)	C28—C29—H29B	108.8 (11)
C11—C12—H12B	109.0 (10)	C30—C29—H29B	109.9 (10)
C13—C12—H12A	108.7 (10)	C28—C29—H29A	107.5 (9)
C11—C12—H12A	107.2 (10)	C30—C29—H29A	110.4 (9)
H12B—C12—H12A	108.7 (14)	H29B—C29—H29A	106.7 (14)
C14—C13—C12	113.87 (14)	C31—C30—C29	114.08 (14)
C14—C13—H13B	109.3 (10)	C31—C30—H30B	109.7 (10)
C12—C13—H13B	107.6 (10)	C29—C30—H30B	107.2 (9)
C14—C13—H13A	109.1 (10)	C31—C30—H30A	110.2 (10)
C12—C13—H13A	107.7 (11)	C29—C30—H30A	106.7 (10)
H13B—C13—H13A	109.2 (14)	H30B—C30—H30A	108.7 (14)
C15—C14—C13	113.89 (15)	C30—C31—C32	113.01 (15)
C15—C14—H14B	110.5 (10)	C30—C31—H31B	109.7 (10)
C13—C14—H14B	107.4 (10)	C32—C31—H31B	107.8 (10)
C15—C14—H14A	108.6 (11)	C30—C31—H31A	108.8 (12)
C13—C14—H14A	107.0 (11)	C32—C31—H31A	108.9 (11)
H14B—C14—H14A	109.4 (15)	H31B—C31—H31A	108.6 (16)
C16—C15—C14	113.34 (16)	C31—C32—H32C	110.0 (12)
C16—C15—H15B	110.3 (12)	C31—C32—H32B	112.1 (12)
C14—C15—H15B	109.3 (12)	H32C—C32—H32B	106.6 (17)
C16—C15—H15A	108.5 (11)	C31—C32—H32A	113.5 (12)
C14—C15—H15A	109.2 (11)	H32C—C32—H32A	106.9 (16)
H15B—C15—H15A	106.0 (17)	H32B—C32—H32A	107.3 (17)

### Hydrogen-bond geometry (Å, °)

**Table:**

*D*—H···*A*	*D*—H	H···*A*	*D*···*A*	*D*—H···*A*
N2—H2N···O3^i^	0.873 (18)	2.234 (19)	3.101 (2)	171.7 (16)
N4—H4N···O1^ii^	0.869 (19)	2.265 (19)	3.121 (2)	168.3 (17)

Symmetry codes: (i) −*x*+1, *y*+1/2, −*z*+1/2; (ii) −*x*, *y*−1/2, −*z*−1/2.
